# elPrep: High-Performance Preparation of Sequence Alignment/Map Files for Variant Calling

**DOI:** 10.1371/journal.pone.0132868

**Published:** 2015-07-16

**Authors:** Charlotte Herzeel, Pascal Costanza, Dries Decap, Jan Fostier, Joke Reumers

**Affiliations:** 1 Imec, Leuven, Belgium; 2 Intel Corporation, Leuven, Belgium; 3 Department of Information Technology, Ghent University—iMinds, Ghent, Belgium; 4 Janssen Research & Development, a division of Janssen Pharmaceutica NV, Beerse, Belgium; 5 ExaScience Life Lab, Leuven, Belgium; CNRS UMR7622 & University Paris 6 Pierre-et-Marie-Curie, FRANCE

## Abstract

elPrep is a high-performance tool for preparing sequence alignment/map files for variant calling in sequencing pipelines. It can be used as a replacement for SAMtools and Picard for preparation steps such as filtering, sorting, marking duplicates, reordering contigs, and so on, while producing identical results. What sets elPrep apart is its software architecture that allows executing preparation pipelines by making only a single pass through the data, no matter how many preparation steps are used in the pipeline. elPrep is designed as a multithreaded application that runs entirely in memory, avoids repeated file I/O, and merges the computation of several preparation steps to significantly speed up the execution time. For example, for a preparation pipeline of five steps on a whole-exome BAM file (NA12878), we reduce the execution time from about 1:40 hours, when using a combination of SAMtools and Picard, to about 15 minutes when using elPrep, while utilising the same server resources, here 48 threads and 23GB of RAM. For the same pipeline on whole-genome data (NA12878), elPrep reduces the runtime from 24 hours to less than 5 hours. As a typical clinical study may contain sequencing data for hundreds of patients, elPrep can remove several hundreds of hours of computing time, and thus substantially reduce analysis time and cost.

## Introduction

**Fig 1:**
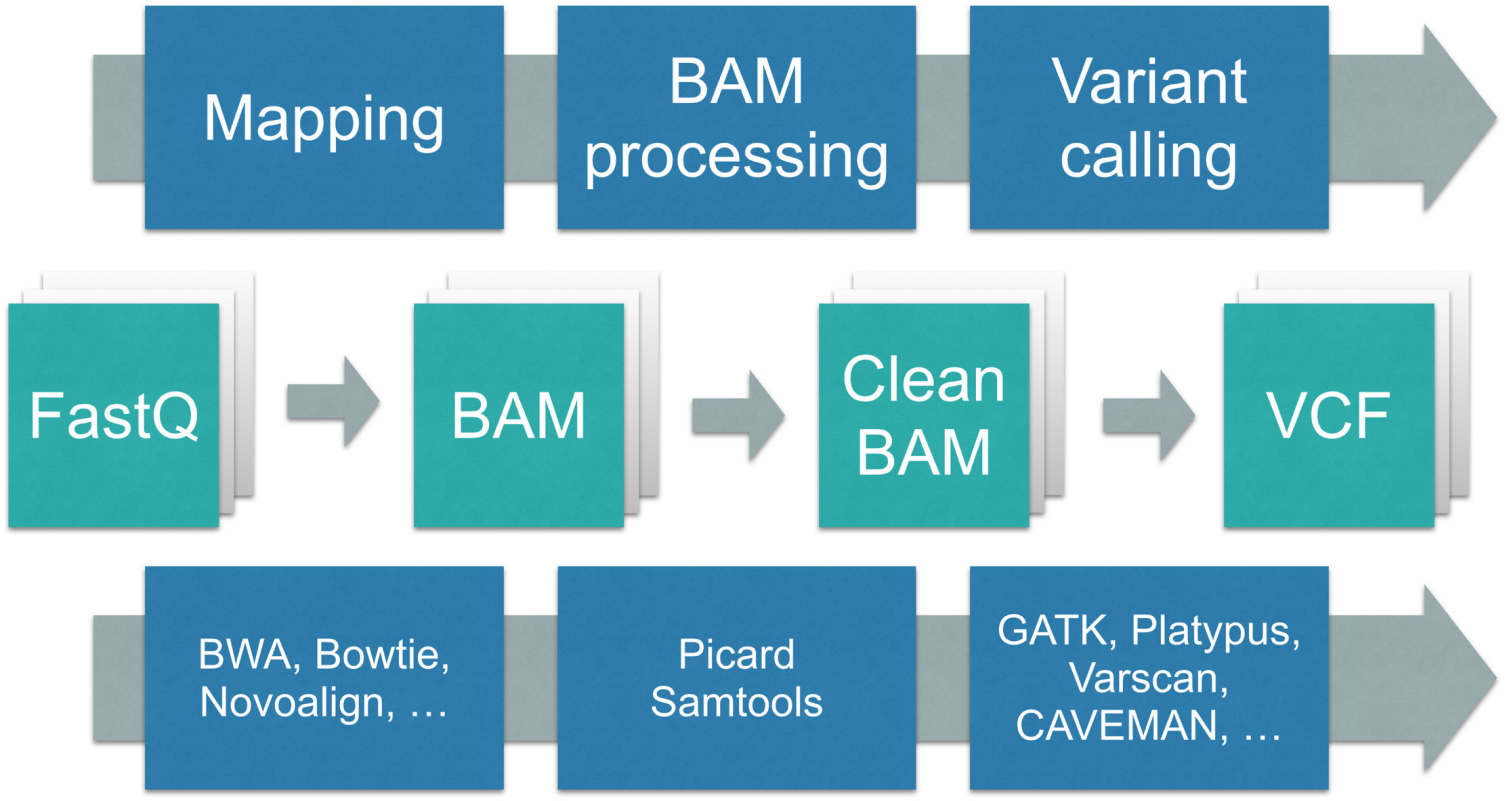
The computational phases of DNA sequencing. First, the reads produced by the wet lab (in FastQ format) are aligned against a reference genome, producing a sequence alignment map file (SAM). Then this SAM file is processed so that it can be used by an analysis tool to produce a VCF file.

DNA sequence analysis generally consists of a mapping phase followed by an analysis phase (Fig 1). In the mapping phase, the reads sequenced in the wet lab are mapped to a known reference genome via an alignment tool, such as BWA [1]. Afterwards, the mapped reads are processed by an analysis tool, for example for variant detection, such as GATK [2]. A large variety of alignment and analysis tools exist, each with their specific use cases.

Alignment and analysis tools communicate via sequence alignment/map (SAM) files, a standardised file format for storing mapped reads [3], or the compressed variants thereof (BAM/CRAM) [4, 5]. A SAM file is a tab-separated file that stores information about the reads generated by the sequencer, such as their query template names and their segment sequences, as well as information generated by the alignment tool, for example the positions where the reads map to the reference genome, and the CIGAR strings that describe how well the reads map to these positions [6]. The SAM format is a very flexible semi-structured format that allows storing optional and tool-specific information.

**Fig 2:**
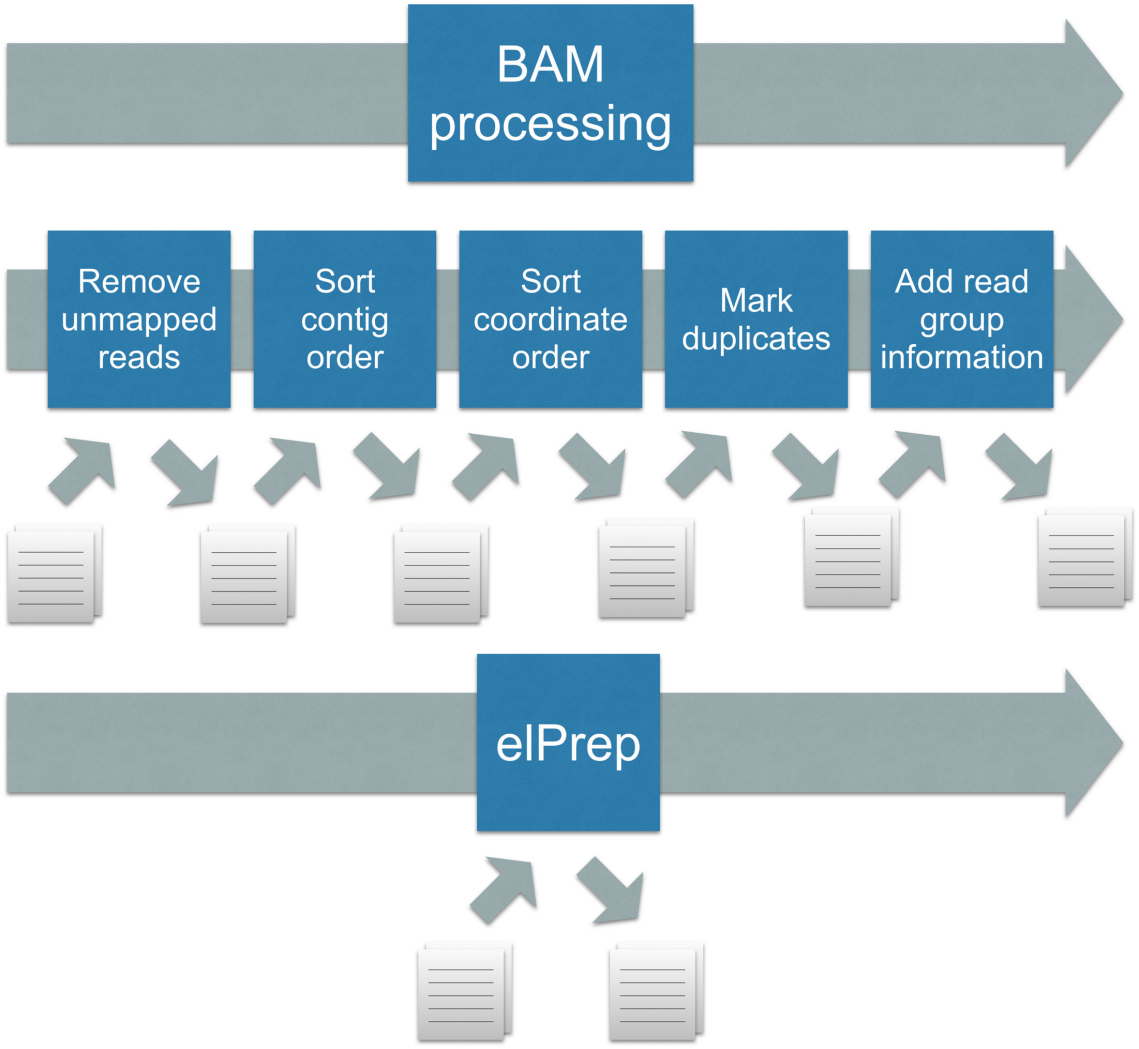
BAM processing: standard practice (top) versus elPrep (bottom). The standard practice is calling a (different) preparation tool for each step, which leads to repeated file I/O, as well as repeated traversal of the same SAM file. To use elPrep, one instead issues a single command that lists the preparation steps to be applied to a SAM file. elPrep internally combines the execution of the different preparation steps, resulting in a single pass over the SAM file, and avoiding repetitive file I/O.

In practice, different alignment tools produce slightly different outputs, and different analysis tools depend on slightly different SAM structures to work properly. For example, some analysis tools require optional information to be present, or require the reads to be filtered, for example to remove unmapped reads, or only work if the reads are stored in a particular order, and so on. This is why in practice, there are typically a number of steps in between the alignment and analysis tools to rewrite the SAM file into a form that is accepted by the analysis tool (Fig 2). For example, the GATK Best Practices [7] and the bcbio-nextgen project [8] give recommendations on which SAM manipulation tools need to be called to successfully combine different alignment and analysis tools.

SAMtools [3] and Picard (http://picard.sourceforge.net/) are arguably the most widely used tools for manipulating SAM files. They are command-line tools with commands for sorting and filtering reads, for adding optional information, for marking polymerase chain reaction (PCR) duplicates based on mapping positions, and so on. A pipeline script calls several of these commands one after the other, each call creating an intermediate SAM file, to eventually end up with a SAM file that is passed as input to the analysis tool.

The computation time spent on preparation steps is not negligible. For example, running a five-step preparation pipeline used at Janssen Pharmaceutica on a whole-exome BAM file takes about 1:40 hours on a standard 24-core server. Since a typical clinical experiment easily consists of several hundreds of BAM files to process, the compute time spent on preparation steps easily adds up to a couple of hundred hours, which incurs a significant waiting time and/or a significant cost, for example for renting the necessary compute nodes in the cloud. The computational challenge for whole-genome data is even more pressing, as BAM files are ten to twenty times larger than exome files. We show that there are opportunities to redesign the software to drastically reduce this runtime and the associated costs.

### Problem statement

The standard practice of creating preparation pipelines by calling multiple command line tools one after the other, has the following drawbacks from a performance perspective (Fig 2):

1. There is repeated file I/O between the steps, including BAM/CRAM compression/decompression, as each command line invocation generates a new SAM file.
2. There are multiple traversals of the same incrementally modified data, as each preparation tool iterates over entire SAM files representing that data to perform its particular computation.
3. Parallelisation opportunities are limited as each tool invocation introduces a synchronisation point.

We propose a software architecture where the execution of a preparation pipeline, independent of which preparation steps are used, requires only a single pass through the SAM file. We have implemented this architecture in the form of a concrete tool called *elPrep*. With elPrep, a user issues only a single command that lists all preparation steps to be applied. The software internally takes care of merging and parallelising the execution of the different steps. This eliminates the end user’s need for naming and organising the storage for intermediate files, for understanding advanced concepts like Unix pipes and when they are applicable or not, and ultimately reduces the runtime and the associated costs.

## Implementation

elPrep is developed and maintained at the ExaScience Life Lab (http://www.exascience.com) for the Linux operating system. End users either use elPrep directly as a command line tool, or can use Python for scripting. All relevant configuration options are documented and their uses are illustrated with example Python scripts. The core elPrep execution engine is implemented in Common Lisp, and can be compiled either with the commercial LispWorks compiler, or the open-source SBCL compiler, both widely used and actively maintained implementations of Common Lisp. A precompiled binary can be downloaded, along with documentation and source code from the elPrep github repository at http://github.com/ExaScience/elprep, released under a BSD-style open source license, and therefore free for both non-commercial and commercial uses. For developers who wish to extend elPrep, extensive API documentation is also available at http://exascience.github.io/elprep/elprep-package/index.html—however, such detailed information is not needed for regular uses of elPrep.

## Methods

elPrep is designed as a high-performance alternative to existing tools for manipulating SAM, BAM, and CRAM files. The software is designed to run in memory, avoiding repeated file I/O between the preparation steps and merging their computations to execute more efficiently. Additionally, elPrep is designed as a multithreaded program from the ground up, so that *all* preparation steps can be executed in parallel, without any unnecessary barriers in between steps.

### A single-pass, filtering architecture

A key idea behind elPrep is to distinguish between SAM manipulation tools that can be expressed as operations or *filters* that work on individual reads, and operations that affect the whole set of reads such as sorting. A pipeline of SAM manipulation tools that are expressed as filters can be executed using a single loop over the SAM file, as illustrated by the pseudo code in Listing 1. The idea is that the loop makes a single pass over the reads in the SAM file and executes the different filters one after the other on each read it encounters this way. Filters may have side effects, for updating the information stored for the read, and return a Boolean value for checking whether the read is to be included in the output file.

**Listing 1:** Execution of the preparation pipeline as a single loop over the input file.

filters = [remove_unmapped, mark_duplicates, …]
loop **for** read **in** input_file:
flag = true
loop **for** function **in** filters:
flag = flag **and apply**(function, read)
**if** flag:
write read to output_file

From a top-level perspective, elPrep can be viewed as a loop that parses the reads from file into memory, applies the filters on the individual reads, passes the filtered reads to the operations that work on the whole set of reads, and finally writes the reads one by one to the output file. In contrast, the execution of preparation pipelines created by calling multiple command-line tools one after the other, results in a separate loop for each filter operation. Computationally, all these loops are *O*(*n*) operations, so the overall execution differs only by a constant factor. However, since SAM/BAM files are large, this constant has a big impact on the actual runtime. Because elPrep only makes a single pass through the data, this also avoids the repeated file I/O that occurs when combining multiple calls to different tools.

### A parallel architecture

elPrep is designed to take advantage of multithreading for parallel processing. To this end, elPrep defines an *input* thread, *worker* threads, and an *output* thread. The input thread streams the data from the input file into memory, while distributing the reads among the available worker threads. The worker threads execute the preparation steps in parallel that are formulated as filters on the incoming reads, modifying their state. Once all data is streamed into memory and filters are applied, the operations that work on the whole data set, such as sorting, are executed. elPrep implements this phase using fork-join patterns, which are executed on a work-stealing scheduler for load balancing [9]. After the processing phase, the worker threads transform the data back into SAM file entries in parallel, while possibly applying additional filters, to finally send the result to the output thread which writes it to the output file.

In practice, only some commands in existing tools for manipulating SAM files make use of multithreading. One strategy could be to parallelise the codes that implement the different commands. However, the execution strategy in elPrep has the advantage that there is no synchronisation between preparation steps.

### A modular plug-in architecture

To facilitate a modular plug-in architecture, the elPrep execution engine is designed as a collection of *higher-order functions*, and filters are implemented as *lambda expressions*. Lambda expressions are a language feature for implementing anonymous *first-class functions*, functions that can be treated as values, for example by passing them as input parameters, or using them as return values.

Lambda expressions are typically known from *pure* functional programming languages that do not allow for side effects such as assigning new values to object fields. However, lambda expressions are useful also outside of pure functional programming, and have for example been introduced more recently in C++11 (2011) and Java 8 (2014). elPrep ensures through its design that modifications to the header and read objects passed to filters are safe.

Concretely, filters in elPrep are modelled by layering three levels of filtering functions (Listing 2). A filter is implemented as a function that implements or returns a *header filter*. A header filter is a function that receives as input the SAM header it can modify, and possibly returns a *thread-local* filter. The header filter is a global filter that is executed once for processing the SAM file. The variables declared in the header filter are visible by all worker threads. The thread-local filter returned by the header filter is a function that receives no arguments and returns a *read filter*. The body of a thread-local filter can be used to set up thread-local variables that are shared by the invocations of the read filter within a worker thread. The read filter itself is a function that receives a read object it can modify, and returns a Boolean value, indicating whether the read is to be included in the output or not.

**Listing 2:** Skeleton structure of a filter definition in elPrep.

**filter** = **lambda** header:
… *# modify header*
**lambda**:
… *# thread–local variables*
**lambda** read:
… *# modify read alignment*
**return** true **or** false

The pseudo code in Listing 3 illustrates the implementation of a filter for removing unmapped reads. According to the SAM specification [3], a read is unmapped when the third bit of the flag entry of the read is zero. This is checked by the read filter, implemented by the third lambda expression. Since the filter for removing unmapped reads does not modify the header of the SAM file, the body of the header filter is empty (first lambda expression). The filter does not require any thread-local variables, hence the body of the thread-local filter is also empty (second lambda expression).

**Listing 3:** Removing unmapped reads as a filter in elPrep.

filter_unmapped = **lambda** header:
**lambda**:
**lambda** read:
**return** (read . flag & #*x4) == 0*

The advantage of using lambda expressions for implementing filters is that they allow for treating filters in elPrep as modular plug-ins: New filters can be easily added or removed without the need to know about the internal implementation details of the elPrep execution engine or the other filters.

### Expressing duplicate marking in elPrep

Most preparation tools, such as replacing the header, replacing the sequence dictionary, filtering unmapped reads, or replacing read groups, are trivial to express within the elPrep framework. However, some preparation tools require algorithmic reformulations. A non-trivial example is duplicate marking, which is used for identifying PCR duplicates. PCR duplicates occur when the same DNA molecule is read multiple times during the sequencing process in the wet lab. They are hard to identify because PCR duplicates do not necessarily produce the exact same segment sequences. A common approach is to identify PCR duplicates in software after the mapping phase by comparing the reads that map to the same position in the reference genome, and marking the reads with the lowest quality scores as duplicates. It is a computationally intensive process as each read needs to be compared to each other read, which, in general, is an *O*(*n* ^2^) process.

#### Picard algorithm

One of the most widely used duplicate marking algorithms is implemented by the Picard program (http://picard.sourceforge.net/). This tool is recommended when targeting the GATK variant caller [7]. In elPrep, we implement the same algorithm used in Picard in the sense that the output produced by the elPrep algorithm is equivalent to the output produced by the Picard algorithm, yet the structure of the algorithm is different.

The Picard algorithm for duplicate marking is a multi-pass algorithm that reads the input file multiple times. Structurally, there are three phases in the Picard algorithm (pseudo code can be found in our technical presentation at http://www.exascience.com/public-files/elprep). In the first phase, the reads are sorted according to mapping coordinates, while keeping track of the original positions of the reads as they occur in the input file. In a second phase, the algorithm identifies the groups of potential duplicates within the sorted list by grouping together all reads that map to the same position. For each of those groups of reads, the algorithm identifies the read with the highest quality score, while keeping track of the file positions of all other reads in that group. Finally, in the third phase, a new output file is written by copying the reads from the original file, using the file positions identified in the second phase to identify which reads are marked as duplicate.

#### Expressing duplicate marking as a filter

We need to reformulate the multi-pass Picard algorithm as a filter operation to make it fit with the single-pass framework of elPrep. The basic idea is to define a memoization table to keep track of the read with the best quality score for each read position seen so far as the execution progresses (Listing 4). Each time it processes a new read, the algorithm checks the memoization table if a read was already encountered that has the same mapping position, in which case the algorithm marks the read with the worse quality score as a duplicate, and puts the read with the better quality score in the memoization table. Since the memoization table must be shared between worker threads, we use a concurrent hash table implementation to avoid unnecessary contention (not shown in Listing 4).

**Listing 4:** Duplicate marking as a filter in elPrep (simplified).

filter_duplicate = **lambda** header:
cache = []
**lambda**:
**lambda** read:
cached_read = cache . **hash**(read . pos)
**if** read . score > cached_read . score
cached_read . mark()
cache . remove(cached_read)
cache . add(read, read . pos)
**else**:
read . mark()

#### Splitting execution in genomic regions

elPrep provides the option to process preparation pipelines per genomic region, a practice for splitting up sequencing workloads. However, it is not trivial to apply this technique to the Picard algorithm.

Picard considers two reads potential duplicates if they both map to the same genomic region *and* the same position within that region. Paired-end reads are compared by comparing the reads that make up the pairs. One difficulty is that Picard guarantees duplicate marking of *fragments*, reads that are part of a pair where the mate is missing from the SAM file. The algorithm cannot simply look at a read and conclude that its mate is missing. Instead, it has to go through all of the reads in the file to determine which pairs are complete before comparing the fragments among each other. Another reason why processing per genomic region in Picard is hard, is that reads of a pair may not map to a single genomic region, and Picard guarantees the correct duplicate marking of such read pairs.

Since the Picard algorithm is designed to process the SAM file as a whole, simply splitting up the workload to run the Picard algorithm per chromosome or genomic region, changes the outcome, and the Picard program provides no options to run it as such. elPrep allows duplicate marking of SAM files per genomic region without changing the outcome compared to running Picard on the whole file.

**Fig 3:**
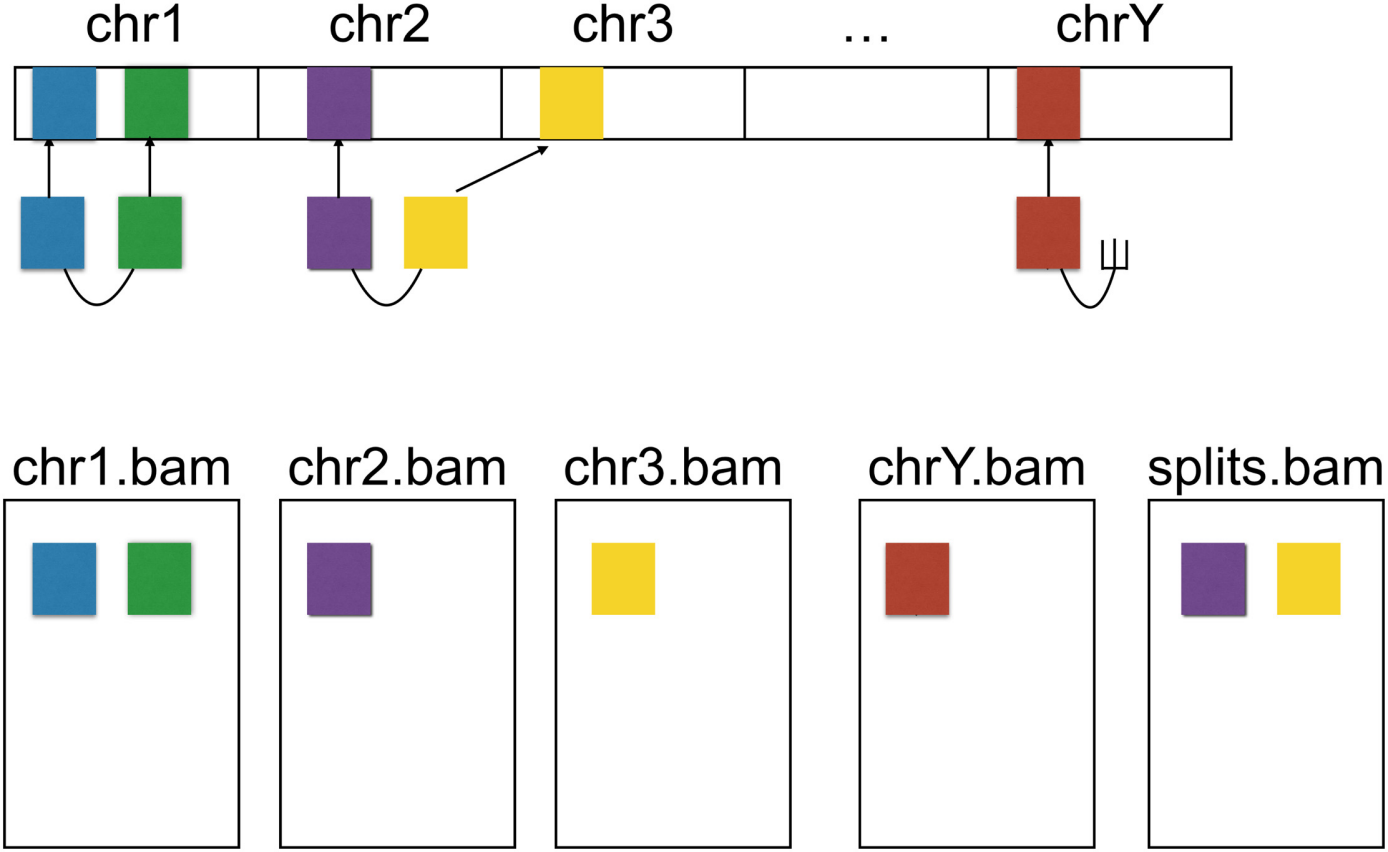
Distribution of read pairs and fragments among split files. elPrep allows splitting SAM files into smaller files which can be processed in parallel, without information loss. The figure shows the mapping of the reads to the reference (top) and shows that elPrep generates a file per genomic region (bottom). Pairs (see chr1) and fragments (see chrY) that map to a single genomic region are put in the files that match those regions. Pairs where reads map to different genomic regions (see reads mapping to chr2 and chr3) are put in a separate split file (far right). The individual reads of those pairs are also duplicated in the files that match the genomic regions where the reads map. This strategy guarantees that all split files contain all information for duplicate marking.

To achieve this, the elPrep algorithm looks at the sequence dictionary in the header of the SAM file to identify the genomic regions, and splits up the SAM file into multiple smaller files, one for each genomic region. Normally, the elPrep splitter simply assigns a read to the file that matches the genomic region to which the read maps. Reads that are part of a pair where the reads map to different genomic regions, are collected in a separate split file. This ensures that such read pairs are complete in that file. The reads where the mate maps to a different genomic region are also duplicated in the file that matches the genomic region where the read maps (Fig 3).

The elPrep splitting strategy guarantees that all split files have all information for correct duplicate marking. The reads of a pair always end up in the same split file, so duplicate marking of pairs can be done separately per split file. As for fragment reads, the fragments that map to the same position, end up in the same split file, so duplicate marking can also be done per split file. The case where Picard marks fragments as duplicates when a read pair exists where one read maps to the same position, is also covered. If the full pair maps to the same genomic region as the fragment, it is just present in the same split file. If the fragment matches a read that is part of a pair that spans different genomic regions, that read was duplicated in the same file by the splitter.

elPrep of course also provides a command for merging the results of processing multiple split files after marking duplicates.

## Results

We claim that elPrep is more efficient than the standard practice of calling multiple command-line tools one after the other. We shows this is mainly because our software architecture requires making only a single pass through a SAM file to execute a preparation pipeline.

### Benchmarks

To prove our claims we set up benchmark experiments for three pipelines for preparing BAM files for variant calling with GATK. The GATK Best Practices recommendations [7] provide guidelines for two preparation pipelines. The first preparation pipeline is part of *Base protocol 1*, a protocol that describes the best practice to go from unaligned FASTQ files to a BAM file that can be used by GATK. This protocol explains how to do alignment with BWA and then discusses two preparation steps:

1. Sorting the BAM file for coordinate order using Picard;
2. Marking the duplicate reads with Picard.

The pseudo code in [7] suggests that both steps can be performed by a single Picard command, but Picard actually requires issuing separate commands for sorting and duplicate marking.

A second preparation pipeline is discussed in [7] (*Support protocol 3*), which is recommended for preparing BAM files that one downloads from online data repositories or receives from colleagues, and may not be properly formatted for GATK. This preparation pipeline consists of:

1. Sorting the BAM for coordinate order with Picard;
2. Marking duplicate reads with Picard;
3. Adding or replacing read groups with Picard.

In practice, it may be necessary to perform additional formatting steps, which is documented in the online documentation of GATK [10], or the domain expert may decide to perform additional filtering steps on the reads before doing the variant calling.

The third preparation pipeline we discuss as a benchmark is the one that is used at Janssen Pharmaceutica (*JP protocol*). Their pipeline to prepare BAM files for variant calling consists of five steps:

1. Sorting the BAM for coordinate order with Picard;
2. Removing unmapped reads and reads with erroneous mapping scores/flags with SAMtools;
3. Marking duplicate reads with Picard;
4. Replacing read groups with Picard;
5. Reordering and filtering the sequence dictionary with Picard.

#### Software and data sets

We execute the three pipelines with both elPrep and Picard/SAMtools. Our goal is to show that, in contrast to existing tools, the execution time with elPrep is largely independent of how many preparation steps need to be executed. We use the latest release of all tools at the time of writing, namely elprep-2.3, samtools-1.2, and picard-tools-1.129. We chose to execute the pipelines with both an exome workload (Illumina high-coverage whole-exome NA12878, human genome [11]) and a whole-genome workload (Illumina Platinum genomes, NA12878, 100bp, 50-fold coverage, human genome [12]).

#### Hardware

All our benchmarks were run on a 24-core server, consisting of two 12-core Intel Xeon E5-2690 processors clocked at 2.6 Ghz, allowing the simultaneous execution of up to 48 hyper-threads. The server is equipped with 256GB RAM and a 2TB Intel P3700 SSD hard disk for storing intermediate files. The machine runs CentOS 7.0 with Linux kernel 3.19.0.

#### Validation

elPrep produces BAM files that are equivalent to those produced by Picard and SAMtools for overlapping functionality. We have verified the equivalence by performing a textual comparison of the BAM files uncompressed to SAM format (essentially using the Unix *diff* command, see S2 Appendix for a detailed discussion).

### Whole-exome benchmark (NA12878)

The Picard versions of the three pipelines consist of scripts that call the individual Picard commands one after the other. There is no composition mechanism in Picard to combine the execution of the different pipeline steps. There is also no support in Picard for using streaming with Unix pipes. Hence the execution times of the full pipelines are equal to the sum of the execution times for the individual steps.

While in elPrep it is possible to build pipelines using separate elPrep commands connected via Unix pipes, we claim it is much more efficient to formulate the pipeline using a single elPrep command that lists all the pipeline steps. We formulated the pipelines using both approaches in elPrep to do the comparison (Tables 1–3). elPrep is flexible in terms of how much RAM it uses via its split/merge tools. We executed elPrep once with giving it access to an amount of RAM that is similar to what Picard uses by splitting the input file per chromosomal regions as they occur in the header of the BAM (third column), and we also did a run with giving elPrep access to all available RAM on the benchmark server (fourth column). All of the benchmark runs we show for elPrep were executed with 48 threads, as were the SAMtools calls. There is no option to configure the number of threads used by Picard.

**Table 1:** Benchmarks of the 2-step pipeline on NA12878 exome (Basic protocol 1).

	Picard		elPrep		elPrep (max RAM)	
	Time	RAM	Time	RAM	Time	RAM
Sort by coordinates	22m 36s	12GB	15m 33s	19GB	10m 5s	180GB
Mark duplicates	31m 19s	23GB	14m 23s	22GB	8m 58s	216GB
Separately executed steps (total)	**53m 55s**	23GB	29m 57s	22GB	19m 3s	216GB
Combined execution steps	na	na	**15m 20s**	22GB	**10m 58s**	216GB

In elPrep, the combined execution of both preparation steps is faster than running the steps one after the other. When elPrep gets to use a similar amount of RAM as Picard uses, it executes the pipeline three times faster than Picard (third column). When elPrep is given access to all available RAM, it executes the pipeline five times faster than Picard (fourth column).

**Table 2:** Benchmarks of the 3-step pipeline on NA12878 exome (Support protocol 3).

	Picard		elPrep		elPrep (max RAM)	
	Time	RAM	Time	RAM	Time	RAM
Sort by coordinates	22m 36s	12GB	15m 33s	19GB	10m 5s	180GB
Mark duplicates	31m 19s	23GB	14m 23s	22GB	8m 58s	216GB
Add read groups	22m 55s	0.6GB	15m 23s	1.7GB	6m 20s	2.7GB
Separately executed steps (total)	**76m 50s**	23GB	45m 20s	22GB	25m 23s	216GB
Combined execution steps	na	na	**15m 47s**	23GB	**10m 34s**	219GB

In elPrep, the combined execution of all three preparation steps is faster than running the steps one after the other. Compared to Basic protocol 1 (Table 1), the extra step in this pipeline does not add an additional runtime cost when combining the execution of the steps with elPrep. elPrep executes the full pipeline five to seven times faster than using Picard, depending on how much RAM elPrep can use, namely the same amount as Picard (third column) or all available RAM (fourth column).

**Table 3:** Benchmarks of the 5-step pipeline on NA12878 exome (JP protocol).

	Picard*/SAMtools^+^		elPrep		elPrep (max RAM)	
	Time	RAM	Time	RAM	Time	RAM
Sort by coordinates	22m 36s*	12GB	16m 4s	19GB	10m 19s	180GB
Filter unmapped reads	3m 16s^+^	0.8GB	14m 58s	1.5GB	6m 12s	2.8GB
Mark duplicates	30m 47s*	23GB	14m 18s	22GB	8m 48s	216GB
Add read groups	22m 39s*	0.7GB	14m 49s	1.7GB	6m 42s	2.5GB
Filter sequence dictionary	20m 39s*	11.8GB	14m 48s	19GB	9m 35s	189GB
Separately executed steps (total)	**99m 58s**	23GB	74m 56s	22GB	41m 37s	216GB
Combined execution steps	na	na	**15m 31s**	23GB	**10m 23s**	219GB

In elPrep, the combined execution of all preparation steps is faster than running the steps one after the other. The execution of the five-step pipeline in elPrep does not take significantly longer than the execution of the two-step and three-step pipelines (Tables 2 and 3). elPrep executes the full pipeline between six times faster when using a similar amount of RAM as Picard (third column) and ten times faster when given access to all available RAM (fourth column).

For the two-step pipeline (Table 1), the combined execution of the steps with a single elPrep invocation is almost two times faster than executing the steps as separate elPrep commands. In case of the three-step (Table 2) and five-step (Table 3) pipelines, the combined execution in elPrep is respectively three and five times faster. In terms of total runtime, there is not much difference between executing the two-step, three-step or five-step pipeline in elPrep. In contrast, there is a significant difference between the runtimes for the Picard versions of the pipelines. The three-step pipeline is 1.5 slower than the two-step pipeline, and the five-step pipeline is almost two times slower.

For a direct comparison between elPrep and Picard, we also need to compare the execution times of the individual steps. We see that the elPrep versions are typically between a factor 1.5 and 2 faster than the equivalent Picard versions. However, the main performance advantage of elPrep comes from its ability to merge the execution of multiple commands. Using this functionality improves the performance for the execution of the full pipeline by another factor two, three, and five for respectively the two-step, three-step, and five-step pipelines. Overall, elPrep executes the two-step pipeline three to five times faster, the three-step pipeline five to seven times faster, and the five-step pipeline six to ten times faster than Picard.

In practice, a clinical experiment consists of 300 or more samples to process, and using elPrep thusly saves several hundred hours of computing time, namely 200+ hours for the two-step pipeline, 300+ hours for the three-step pipeline, and more than 450 hours for the five-step pipeline.

### Whole-genome benchmark (NA12878)

We executed the same three preparation protocols on the whole-genome data set for NA12878 (Tables 4–6). Similar to what we see for the exome benchmarks, the combined execution of the different preparation steps in elPrep is more efficient than executing the steps one after the other.

**Table 4:** Benchmarks of the 2-step pipeline on NA12878 whole genome (Basic protocol 1).

	Picard		elPrep	
	Time	RAM	Time	RAM
Sort by coordinates	5h 14m	12GB	5h 2m	203GB
Mark duplicates	7h 45m	28GB	5h 16m	234GB
Separately executed steps (total)	**12h 59m**	28GB	10h 18m	234GB
Combined execution steps	na	na	**5h 9m**	239GB

Similar as with exome data, the combined execution of the preparation pipeline in elPrep is much faster than executing the individual steps one by one, which is the only option with Picard. elPrep executes Basic protocol 1 about 2.5 times faster than Picard.

**Table 5:** Benchmarks of the 3-step pipeline on NA12878 whole genome (Support protocol 3).

	Picard		elPrep	
	Time	RAM	Time	RAM
Sort by coordinates	5h 14m	12GB	5h 2m	203GB
Mark duplicates	7h 45m	28GB	5h 16m	234GB
Add read groups	6h 51m	0.7GB	1h 26m	20GB
Separately executed steps (total)	**19h 51m**	28GB	11h 44m	234GB
Combined execution steps	na	na	**5h 11m**	239GB

The execution time with elPrep remains stable compared to Basic protocol 1 (Table 4). elPrep executes this preparation pipeline almost four times faster than Picard.

**Table 6:** Benchmarks of the 5-step pipeline on NA12878 whole genome (JP protocol).

	Picard*/SAMtools^+^		elPrep	
	Time	RAM	Time	RAM
Sort by coordinates	5h 14m*	12GB	5h 2m	203GB
Filter unmapped reads	42m 16s^+^	0.8GB	1h 26m	2.5GB
Mark duplicates	7h 4m*	29GB	5h 1m	233GB
Add read groups	5h 18m*	0.8GB	1h 18m	2.8GB
Filter sequence dictionary	5h 6m*	12GB	4h 54m	196GB
Separately executed steps (total)	**23h 25m**	29GB	17h 42m	233GB
Combined execution steps	na	na	**4h 47m**	239GB

Again, combined execution in elPrep is faster than executing the steps one by one. elPrep executes the five-step pipeline faster than the three-step pipeline (Table 5), even though it has two more steps. This is because the second step here removes unmapped and erroneously tagged reads. This reduces the number of reads that are processed by the subsequent steps. This can be seen by looking at the timings of the individual steps, both for elPrep and Picard, as well. Overall, elPrep executes the five-step pipeline almost five times faster.

We executed the benchmarks by having elPrep split up the input file by chromosomal regions as they occur in the header of the BAM file. elPrep uses an amount of RAM that is proportional to the input size of the split files, which, in the case of the whole-genome benchmarks, means that elPrep uses 8.5× more RAM than Picard/SAMtools (see the RAM entries in Tables 4–6).

A solution to use less RAM would be to use a more fine-grained splitting strategy than splitting by chromosomal regions, but elPrep currently does not support this. If machines with sufficient RAM are not available to the user, we recommend other tools that are optimised for low-memory footprints (discussed in detail in S1 Appendix) or switching to a cloud-based solution. For example, when using Amazon Web Services, the cost of renting a memory-optimised server that runs elPrep is in the same range as renting a server with less RAM that runs Picard/SAMtools (https://aws.amazon.com/ec2/pricing/ as of May 19, 2015). Based on the resource requirements in Tables 4–6), we need to run elPrep on an r3.8xlarge (memory optimised) instance with 32 virtual CPU cores, 244GB RAM, and 2× 320 GB SSD drives, which costs $2.800/hour. For running the Picard/SAMtools pipelines, a i2.xlarge (storage optimised) instance with 4 virtual CPU cores, 30.5GB RAM, and 1× 800 GB SSD drive would suffice, which costs $0.853/hour. Hence the instance for running the pipelines with elPrep costs roughly 3.3× more than the instance for using Picard/SAMtools. However, since elPrep executes the pipelines between 2.5 to 5× faster than Picard/SAMtools (see Tables 4–6), the server cost of using elPrep is in the same range for Basic protocol 1, and cheaper for Support Protocol 3 and the JP Protocol, than using Picard/SAMtools, with the added benefit of a substantially reduced waiting time for the elPrep user.

## Related Work

There is a large body of related work that focuses on optimising individual SAM manipulation tools (see S1 Appendix for a detailed overview). For example, many tools focus on optimising memory use of duplicate marking, for example by defining data structures that overflow to disk when a certain threshold is reached (bamUtil [13], biobambam [14], Sambamba [15]) or define an alternative duplicate marking strategy that does not require comparing all alignments (SAMBLASTER [16]). Some of these tools are faster for executing the individual tools they implement, or use substantially less RAM than elPrep. However, none of the tools offer a way of combining the execution of multiple tools like elPrep does. Whereas we focus on tackling the drawbacks of composing pipelines as separate command invocations, the related work focuses on optimising individual tools, such as duplicate marking and sorting for coordinate order. These results are orthogonal, and it should be possible to add the optimisations to elPrep, or to redesign the other tools to use a software architecture similar to that of elPrep. Since lambda expressions have recently been added to languages such as C++11 and Java 8, such a redesign should be viable for at least some of the related work.

Other related work focuses on integrating complete pipelines into single applications with a focus on taking advantage of computational resources, like BALSA [17], optimised for GPUs, and ISAAC [18], optimised for servers with high amounts of RAM. Since these tools have full control over the pipeline, they have more opportunities for optimisations, such as defining their own alignment format instead of using the generic SAM format (BALSA), or partially combining secondary analysis steps such as filtering with the alignment phase (ISAAC). However, an advantage of using de-facto, community-driven standard file formats like SAM/BAM/CRAM is that they provide scientists the freedom to freely choose different tools from different tool authors for different phases of the pipeline. This is also partially acknowledged by the integrated pipeline solutions, in that they are also split into subtools: ISAAC comes with a separate aligner and variant caller and is open source, so that modifications to these tools are in principle possible; BALSA, while closed source, provides *snapshot files*, its own format for representing alignments, which can be used to implement one’s own secondary analysis and variant caller tools. Unlike these other integrated tools, elPrep focuses on modifying SAM/BAM/CRAM files to ease the combined use of different aligners and variant callers.

## Conclusions and Future Work

The main contribution of this paper is a software architecture for SAM/BAM manipulation tools that only requires a single pass through a SAM file to execute a sequencing pipeline, independent of which and how many tools need to be applied. The idea is to define the execution engine in terms of higher-order functions and define the manipulation tools as lambda expressions, allowing for a modular design where individual tools are implemented without a need to know how the other tools are implemented or how they are executed (in parallel) by the execution engine.

We have implemented this architecture as a tool called elPrep. With elPrep, a user speficies a sequencing pipeline as a single command, and elPrep takes care of merging and parallelising the execution of the different steps within the pipeline. Our benchmarks show this is much faster than executing the steps one after the other by using separate command invocations, which is the standard practice for defining sequencing pipelines today. elPrep avoids the repeated file I/O that occurs between seperate command invocations, as well as the repeated traversals of the same SAM files, and the explicit synchronisation that limits parallelism with separate command invocations.

Concretely, elPrep executes a five-step pipeline used at Janssen Pharmaceutica between six to ten times faster—depending on how much memory elPrep is allowed to use—than using a combination of SAMtools and Picard invocations. Similarly, elPrep executes a two-step and three-step pipeline from the GATK Best Practices recommendations between respectively three to five times and five to seven times faster than Picard. For exome workloads this means elPrep reduces the runtime from about 1:40 hours to about 10 to 15 minutes, saving hundreds of hours of computation time in a typical clinical experiment. The benefit would be even larger for whole-genome data, where SAM files are ten to twenty times larger.

A possible drawback of elPrep is that it is currently under development, and more mature tools such as SAMtools and Picard are more feature complete.

elPrep focuses on preparation tools for SAM/BAM/CRAM files. We think it should be possible to further optimise the whole sequencing pipeline to take advantage of the single-pass execution strategy of elPrep. For example, elPrep relies on a splitting phase that takes the BAM file and splits it up according to genomic regions for parallel processing. However, we could adapt the aligner tool to directly output multiple split files instead of writing a single BAM file. While this is a minor modification to the aligner, we would avoid the cost of splitting up the BAM file in elPrep. We have not yet explored this in detail.

Another feature of our architecture that we did not discuss in this paper, is that our architecture is agnostic with regard to where input, intermediate, and output data are stored. The framework is designed in such a way that data can reside in files on disk, in memory, or even in a database. For example, preliminary support for MongoDB is provided as a separate extension library for elPrep. Such a feature is interesting for connecting interactive applications to sequencing pipelines, but we need to further explore the impact on performance of our approach.

elPrep can be used as a plugin for the Halvade MapReduce framework for executing sequencing pipelines in parallel on a cluster [19]. This is particularly interesting for further parallelising the execution of whole-genome data.

## Supporting Information

S1 Appendix Detailed overview of related work. Benchmarks and discussion of bamUtil, biobambam, Sambamba, SAMBLASTER, and SAMtools, compared to elPrep.
(PDF)

S2 Appendix Validation of elPrep output compared to Picard/SAMtools output. Detailed comparison of elPrep and Picard/SAMtools output using textual comparison (*diff*).
(PDF)
