# Supplementary material for: elPrep: High-Performance Preparation of Sequence Alignment/Map Files for Variant Calling
Source: PLoS One. 2015 Jul 16;10(7):e0132868. doi: 10.1371/journal.pone.0132868 (PMC4504710; doi:10.1371/journal.pone.0132868)
Supplement: S1 Appendix — Benchmarks and discussion of bamUtil, biobambam, Sambamba, SAMBLASTER, and SAMtools, compared to elPrep. (PDF) [file pone.0132868.s001.pdf]

S1 Appendix: Detailed overview of related work

Charlotte Herzeel[1,5,◑,*], Pascal Costanza[2,5,◑], Dries Decap[3,5], Jan Fostier[3,5], Joke Reumers[4,5]

1 Imec, Leuven, Belgium
2 Intel Corporation, Leuven, Belgium
3 Department of Information Technology, Ghent University - iMinds, Ghent, Belgium
4 Janssen Research & Development, a division of Janssen Pharmaceutica NV, Beerse, Belgium
5 ExaScience Life Lab, Leuven, Belgium

◑These authors contributed equally to this work.
*** Charlotte.Herzeel@imec.be**

Related work – details

Whereas the main focus of elPrep is the performance of the combined execution of all the steps in a preparation pipeline, there are a number of related projects that focus on optimising the performance of individual preparation tools. We benchmarked these tools for the steps in the preparation pipelines that they implement on the exome data set (NA12878) (Tables 1 and 2). We used the latest versions of these tools at the time of writing, namely samtools-1.2, sambamba_v0.5.1, samblaster-v.0.1.21, biobambam-0.0.189, bamUtil v1.0.13.

SAMBLASTER [1] is a tool for duplicate marking that is designed to work as a streaming tool. It proposes a new algorithm where the idea is to mark all reads that have the same mapping position as a previously encountered read as duplicates. Whereas the Picard algorithm keeps the read with the *best* quality score per coordinate, the SAMBLASTER algorithm keeps the *first* read it encounters per coordinate. The outcome of SAMBLASTER therefore differs from Picard and elPrep.

We compared the execution time of elPrep and SAMBLASTER for our exome data set (Table 2). SAMBLASTER expects the reads in the input file to be grouped by queryname, so that it does not need to identify which reads belong to which pair like the Picard algorithm. The idea is to use SAMBLASTER with an aligner that produces such output. Since SAMBLASTER only supports reading and writing of SAM files, it is necessary to use another tool for converting between formats. For our benchmarks, we used SAMtools to convert the input from BAM to SAM and the output from SAM to BAM (also included in the timings in Table 2). SAMBLASTER supports streaming in and out of the tool with Unix piping.

Sambamba (`https://github.com/lomereiter/sambamba`) is a high-performance tool for working with SAM and BAM files. It implements a subset of SAMtools and focuses on multithreading optimisations. Sambamba implements two operations that elPrep also implements, namely duplicate marking and sorting. Sambamba implements a multi-pass duplicate marking algorithm similar to the Picard algorithm and uses temporary files for storing intermediate data. The sort algorithm is a merge sort that operates on disk. Sambamba does not support streaming for those operations. We

benchmarked the sorting and duplicate marking tools of Sambamba on our exome data set (Table 1). We notice that for these two operations, Sambamba is faster than elPrep, even for the combined execution of the two steps, albeit Sambamba uses more RAM overall.

Biobambam [2] is a collection of multithreaded command-line tools for manipulating BAM files. Biobambam focuses on keeping the memory footprint of its tools low. It introduces a layered data structure for identifying read pairs in a BAM file. Briefly, this data structure consists of a hash table, which overflows to a list data structure, which in turn overflows to temporary files on disk once a certain memory threshold is reached. The authors of biobambam show that the Picard duplicate marking algorithm can be expressed using the layered data structure, so that it can run in a very small memory footprint.

Our benchmarks show that biobambam uses the least memory of all approaches for sorting, and only uses more memory than bamUtil for duplicate marking (Table 1). The duplicate marking algorithm of biobambam requires the input to be sorted by coordinate order, so to know the time for duplicate marking, we must also take into account the time for sorting. Biobambam takes the standard approach of implementing each preparation tool as a separate command-line tool, assuming preparation pipelines are created by calling multiple such commands. Biobambam supports streaming with Unix pipes between some of its tools and offers a form of internal streaming without going through Unix pipes as an option for combining sorting and duplicate marking. Overall, the combined execution of sorting and duplicate marking is faster than the combined execution of those steps in elPrep (Tables 1 and 2).

Although the execution time of the sorting and duplicate marking steps are faster in both Sambamba and biobambam, the important advantage of elPrep is that adding more preparation steps does not cost a lot more in terms of execution time when using elPrep. The three-step and five-step pipelines run in roughly the same time as running only duplicate marking and sorting steps (Table 2). This is because elPrep is designed with a single-pass architecture in mind. It is not obvious that we would get a similar behaviour when adding more preparation steps to Sambamba and biobambam. These tools use a model of calling multiple command-line tools one after the other, so when executing multiple preparation steps, they will have repeated file I/O between the steps, repeated traversals of the same data set, and limited parallel throughput as each step introduces a synchronisation point.

BamUtil (`https://github.com/statgen/bamUtil`) is a collection of single-threaded BAM manipulation tools that focus on low memory footprint. When comparing elPrep and bamUtil for the tools that both approaches implement, we see that bamUtil generally uses less RAM than elPrep, albeit elPrep is generally three times faster (Table 2). BamUtil provides no way of combining its tools, so the cost of executing a pipeline is the sum of the runtimes for the individual steps.

It is not possible to compare the related tools for executing the full pipelines, since they only implement certain steps. We can get an idea of what the best pipeline using related tools would be by combining them for speed (Table 3) or for RAM use (Table 4). We are aware that there are other concerns that may be more important for choosing tools. For example, from a biological point of view, the outcome of the underlying duplicate marking algorithm may be more important. In this regard, elPrep guarantees the same outcome as the equivalent Picard pipelines (from the GATK Best Practices recommendations). Another concern may the increased use of disk space with tools that store intermediate runtime data on disk. We refer to the publications and documentation of the related tools for detailed information. Our main interest with this experiment is to give an idea of what the best performance would be today for the three presented pipelines.

Table 1. Benchmarks related work on NA12878 exome (Part 1).

	SAMtools		biobambam		Sambamba	
	Time	RAM	Time	RAM	Time	RAM
1. Sort by coordinates	9m 35s	41GB	2m 12s	1.1GB	1m 51s	38GB
2. Filter unmapped reads	3m 43s	0.4GB	na	na	na	na
3. Mark duplicates	na	na	5m 17s	0.5GB	5m 54s	4.4GB
4. Add read groups	na	na	na	na	na	na
5. Filter sequence dictionary	na	na	na	na	na	na
Basic protocol 1 (steps 1,3)	na	na	**7m 32s**	1.1GB	**7 m 45s**	38GB
Support protocol 3 (steps 1,3,4)	na	na	na	na	na	na
JP protocol (steps 1-5)	na	na	na	na	na	na

Comparison of preparation steps implemented by SAMtools, biobambam, and Sambamba. Biobambam supports composition of the two steps using internal piping. Biobambam focuses on low RAM footprints and makes use of intermediate files.

Table 2. Benchmarks related work on NA12878 exome (Part 2).

	BamUtil		SAMBLASTER		elPrep	
	Time	RAM	Time	RAM	Time	RAM
1. Sort by coordinates	na	na	na	na	-	-
2. Filter unmapped reads	na	na	na	na	-	-
3. Mark duplicates	28m 9s	0.5GB	7m 8s	1.1GB	-	-
4. Add read groups	23m 9s	0.004GB	na	na	-	-
5. Filter sequence dictionary	na	na	na	na	-	-
Basic protocol 1 (steps 1,3)	na	na	na	na	**15m 20s**	22GB
Support protocol 3 (steps 1,3,4)	na	na	na	na	**15m 47s**	23GB
JP protocol (steps 1-5)	na	na	na	na	**15m 31s**	23GB

Comparison of preparation steps implemented by bamUtil, SAMBLASTER, and elPrep. BamUtil has the smallest memory footprint of all tools (for the steps it implements) and relies on intermediate files for storing data. SAMBLASTER implements an alternative duplicate marking algorithm with a low-memory footprint, but its outcome differs from all other tools. Neither tools support a composition mechanism for multiple steps as elPrep does.

Table 3. Benchmarks for a combination of tools, optimized for speed, versus elPrep.

	Optimized for Speed			elPrep	
	Tool	Time	RAM	Time	RAM
1. Sort by coordinate, mark duplicates	biobambam	7m 32s	1.1GB	-	-
2. Filter unmapped reads	SAMtools	3m 43s	0.4GB	-	-
3. Add read groups	Picard	22m 55s	0.6GB	-	-
4. Filter sequence dictionary	Picard	20m 39s	12GB	-	-
Basic protocol 1 (step 1)		**7m 32s**	1.1GB	**15m 20s**	22GB
Support protocol 3 (steps 1,3)		**30m 27s**	1.1GB	**15m 47s**	23GB
JP protocol (steps 1-4)		**54m 49s**	12GB	**15m 31s**	23GB

Timings for the three presented pipelines when building them as a composition of existing tools, optimized for speed. elPrep remains stable across pipelines both in terms of execution time and memory use.

The benchmarks for the related tools selected for speed (Table 3) show that elPrep is around two times slower than a combination of related tools for executing Basic protocol 1. When more steps are added to the preparation pipeline, the execution time with elPrep remains stable. elPrep executes Support protocol 3 roughly two times faster than the fastest combination of related tools, and executes the JP protocol 3.5 times faster.

The experiment to compose the related tools selected for smallest memory footprint (Table 3) shows that elPrep consumes more memory for all three pipelines. It is in

Table 4. Benchmarks for a combination of tools, optimized for RAM, versus elPrep.

	Optimized for RAM			elPrep	
	Tool	Time	RAM	Time	RAM
1. Sort by coordinate, mark duplicates	biobambam	7m 32s	1.1GB	-	-
2. Filter unmapped reads	SAMtools	3m 43s	0.4GB	-	-
3. Add read groups	bamUtil	23m 9s	0.004GB	-	-
4. Filter sequence dictionary	Picard	20m 39s	12GB	-	-
Basic protocol 1 (step 1)		**7m 32s**	1.1GB	**15m 20s**	22GB
Support protocol 3 (steps 1,3)		**30m 41s**	1.1GB	**15m 47s**	23GB
JP protocol (steps 1-4)		**55m 3s**	12GB	**15m 31s**	23GB

Timings for the three presented pipelines when building them as a composition of existing tools, optimized for RAM. elPrep remains stable across pipelines both in terms of execution time and memory use.

principle possible to use the elPrep split/merge tools to reduce the memory footprint of elPrep, but if very small memory footprints are the main concern, it may be more efficient to use tools with dedicated data structures that overflow to disk such as biobambam and bamUtil.

Summary Some of the related work outperforms both Picard and elPrep for individual steps, notably biobambam, Sambamba, and SAMBLASTER. However, none of the related work provides a generic composition mechanism like elPrep does. Biobambam allows for composing the sort and mark duplicates steps through process-internal piping, but there is no obvious way to compose other steps within the same framework beyond using Unix pipes. SAMBLASTER only ensures that SAM files can be piped as input and output using Unix pipes. The other tools discussed above do not support composition of steps beyond using intermediate files to the best of our knowledge.

References

1. Faust G, Hall I. SAMBLASTER: fast duplicate marking and structural variant read extraction. Bioinformatics. 2014 Sep 1;30(17):2503-5. doi:10.1093/bioinformatics/btu314.

2. Tischler G, Leonard S. biobambam: tools for read pair collation based algorithms on BAM files. Source Code Biol Med. 2014 Jun 20;9:13. doi:10.1186/1751-0473-9-13.
