# Supplementary material for: elPrep: High-Performance Preparation of Sequence Alignment/Map Files for Variant Calling
Source: PLoS One. 2015 Jul 16;10(7):e0132868. doi: 10.1371/journal.pone.0132868 (PMC4504710; doi:10.1371/journal.pone.0132868)
Supplement: S2 Appendix — Detailed comparison of elPrep and Picard/SAMtools output using textual comparison (diff). (PDF) [file pone.0132868.s002.pdf]

S2 Appendix: Validation of the elPrep output

Charlotte Herzeel[1,5,☯,*], Pascal Costanza[2,5,☯], Dries Decap[3,5], Jan Fostier[3,5], Joke Reumers[4,5]

1 Imec, Leuven, Belgium
2 Intel Corporation, Leuven, Belgium
3 Department of Information Technology, Ghent University - iMinds, Ghent, Belgium
4 Janssen Research & Development, a division of Janssen Pharmaceutica NV, Beerse, Belgium
5 ExaScience Life Lab, Leuven, Belgium

☯These authors contributed equally to this work.
*** Charlotte.Herzeel@imec.be**

Validation of the output

When discussing the algorithms behind elPrep, we described that elPrep produces the same results as Picard, despite both programs having substantially different software architectures. The goal of elPrep is to improve the performance without changing the semantics of the original algorithms. For example, while elPrep uses a single-pass strategy for duplicate marking, and Picard uses a multi-pass strategy, the selection criteria for identifying duplicates are exactly the same in both programs. Hence while elPrep and Picard compute differently, they produce the same BAM output.

We can validate that elPrep produces the same results as Picard by doing a textual comparison or *diff* of the BAM files produced by both tools. The idea is to compare the BAM files line by line. However, we need to prepare the BAM files for comparison, as there always are a number of irrelevant differences to be expected between BAM files:

1. The SAM specification specifies *no ordering* for the *optional fields* in the alignment section [1]. To be able to compare two BAM files, one should sort the optional fields.

2. The SAM specification foresees an @PG tag in the header section that records information about the programs used to create and process the BAM file [1], including their names and command-line parameters. Similarly, it also specificies an optional field, the PG tag, that records that information per affected alignment in the alignment section. Obviously, these header and alignment tags necessarily differ when different programs are used to prepare BAM files, and should therefore be removed before comparing two BAM files.

3. The SAM specification specifies that BAM files are sorted by coordinate order by comparing the reference sequence name (RNAME) and mapping position (POS) of the alignments. However, there can be multiple alignments withe the same RNAME and POS, and the SAM specification specifies no additional ordering constraints between such alignments. Hence depending on the sorting algorithm used, or the non-determinism of a parallel sorting algorithm, the alignments with the same RNAME and POS may end up in a slightly different order in different

BAM files, yet both are correctly sorted by coordinate order. Hence before comparing BAM files, one should sort the BAM files by query name, which is unique for each alignment. Checking wether a BAM file is sorted correctly by coordinate order can be done separately by attempting to index it.

4. The Picard algorithm compares alignments for duplicate marking by comparing their (adapted) mapping coordinates and (adapted) quality scores. There can be alignments that have exactly the same (adapted) mapping coordinates and (adapted) quality scores. In this case, either alignment could be marked as a duplicate. The Picard algorithm internally sorts the alignments before performing duplicate marking. Hence in Picard, which alignment is marked as duplicate, depends on a combination of the order of the alignments in the input BAM file and the comparator used for the internal sort. Since elPrep is a parallel implementation, it is non-deterministic which alignment is marked as duplicate when there exists another one with exactly the same (adapted) mapping position and (adapted) quality score. We have added a *deterministic* option to the elPrep filter command for duplicate marking to simulate deterministic behaviour, by always marking the read with the shortest query name. With this option, it is possible to have exactly the same outcome across different elPrep runs for duplicate marking. However, there may still be small differences with Picard runs, as the only way to get the exact same ordering of duplicate marking as Picard in elPrep, would be to mimic its internal sort operations, but that would nullify our performance improvements without any real benefit.

There exists integrated *diff* tools for comparing BAM files that allow control over how the BAM files are compared, see for example the *diff* command of bamUtil (`https://github.com/statgen/bamUtil`). Alternatively, one can transform the BAM files taking into account steps 1 to 4 above, convert to SAM format, and compare them using the Unix *diff* tool for line by line comparison of text files. For example, sorting optional fields (step 1) and removing optional fields (step 2) can be done with the biobambam bamauxsort and bamfilteraux tools respectively [2]. Alternatively, a quick sanity check to compare BAM files is to compare the statistics of the BAM files, for example with the SAMtools flagstat command [3], which enumerates the number of duplicates, number of mapped reads, the number of reads with mate mapped to a different chromosome, and so on.

We have compared the BAM files generated for the three pipelines by Picard/SAMtools this way and found no significant differences. The statistics of the BAM files are exactly the same. There are minor differences between the headers of the BAM files: For those generated with elPrep, the @PG header tag records information about elPrep, whereas it records Picard/SAMtools for the other files. We also saw a difference between the MD5 checksums of the @SQ tags in the headers. These are integer values in hexadecimal notation, which are in lower case for the BAM files generated by Picard, whereas elPrep stores them in upper case following the SAM specification [1]. Since the case used in hexadecimal notation does not impact the represented integer value, this difference is irrevelant. We also see a difference in the @HD VN tag, which records the SAM format version used, which in case of elPrep is the latest, namely version 1.5, whereas Picard still uses version 1.4.

When comparing the alignment sections of the BAM files, we found a difference of 18 alignments out of 127.271.202 alignments in the exome workload. We see that some alignments are marked as duplicate in one file, but not in the other. However, for each alignment that is marked as duplicate in the elPrep file and non-duplicate in the Picard file, we see there is an alignment with exactly the same (adapted) mapping position and (adapted) quality score that is then marked as non-duplicate in the elPrep file and

duplicate in the Picard file (and vice versa). Essentially, in these cases, either alignments could be marked duplicate/non-duplicate (step 5), and there is no real difference between the BAM files.

Since there are no real differences between the BAM files generated by elPrep and Picard, elPrep has no impact on the variant calling phase.

References

1. The SAM/BAM Format Specification Working Group. Sequence Alignment/Map Format Specification. 2015 March 3 [cited 24 March 2015]. Available: http://github.com/samtools/sam-spec.

2. Tischler G, Leonard S. biobambam: tools for read pair collation based algorithms on BAM files. Source Code Biol Med. 2014 Jun 20;9:13. doi:10.1186/1751-0473-9-13.

3. Li H, Hansaker B, Wysoker A, Fennell T, Ruan J, Homer N, et al. The Sequence Alignment/Map format and SAMtools. Bioinformatics. 2009 Aug 15;25(16):2078-9. doi: 10.1093/bioinformatics/btp352.
